# Development of *Coriandrum sativum* Oil Nanoemulgel and Evaluation of Its Antimicrobial and Anticancer Activity

**DOI:** 10.1155/2021/5247816

**Published:** 2021-10-11

**Authors:** Ahmad M. Eid, Linda Issa, Omamah Al-kharouf, Raghad Jaber, Fatima Hreash

**Affiliations:** Department of Pharmacy, Faculty of Medicine and Health Sciences, An-Najah National University, Nablus, State of Palestine

## Abstract

This study is aimed at developing coriander oil into a nanoemulgel and evaluating its antimicrobial and anticancer effects. Coriander (*Coriandrum sativum*) oil was developed into a nanoemulgel by using a self-nanoemulsifying technique with Tween 80 and Span 80. Hydrogel material (Carbopol 940) was then incorporated into the nanoemulsion and mixed well. After this, we evaluated the particle size, polydispersity index (PDI), rheology, antimicrobial effect, and cytotoxic activity. The nanoemulsion had a PDI of 0.188 and a particle size of 165.72 nm. Interesting results were obtained with the nanoemulgel against different types of bacteria, such as *Pseudomonas aeruginosa*, *Klebsiella pneumoniae*, and methicillin-resistant *Staphylococcus aureus* (MRSA), with a minimum inhibitory concentration (MIC) of 2.3 *μ*g/ml, 3.75 *μ*g/ml, and 6.5 *μ*g/ml, respectively. In addition, the half-maximal inhibitory concentration (IC_50_) of the nanoemulgel when applying it to human breast cancer cells (MCF-7), hepatocellular carcinoma cells (Hep3B), and human cervical epithelioid carcinoma cells (HeLa) was 28.84 *μ*g/ml, 28.18 *μ*g/ml, and 24.54 *μ*g/ml, respectively, which proves that the nanoemulgel has anticancer effects. The development of *C. sativum* oil into a nanoemulgel by using a self-nanoemulsifying technique showed a bioactive property better than that in crude oil. Therefore, simple nanotechnology techniques are a promising step in the preparation of pharmaceutical dosage forms.

## 1. Introduction

In the past, natural herbal products have been used by humans to treat many diseases based on their traditions and conventional knowledge. New research indicates that natural compounds can treat major diseases, such as cancer, microbial disease, diabetes, and cardiovascular disease. However, using these natural compounds was limited because of their biocompatibility and toxicity. On the other hand, the large size that these compounds possess causes poor solubility, absorption, and poor precise delivery targets, leading to impairment of their effectiveness [1]. Therefore, using a modern drug delivery method to address these problems might be a good solution for these critical issues [2].

Coriander (*Coriandrum sativum*) is an aromatic, annual herb, mostly growing in the Mediterranean region (specifically in India), that belongs to the *Apiaceae* family, which is considered one of the most often used plants in medicine. Many studies have shown that the coriander plant exhibits several pharmacological actions, including antimicrobial, anticancer, antioxidant, anticonvulsant, cholesterol-lowering activity, and many other actions [3]. Essential oils and fatty acids are considered the major constituents of this herbaceous plant. The coriander plant contains 70.11% of the essential oil linalool (3,7-dimethyl-1,6-octadien-3-ol), which has antibacterial activity. A synergetic effect was reported when added to other antibiotics, such as ciprofloxacin, tetracycline, and amoxicillin. Interestingly, a marked reduction of the minimum inhibitory concentration (MIC) was noticed after combining antibiotics and linalool. This was a promising step in developing a new therapy related to bacterial infections [4]. Linalool is a nontoxic compound, which has many applicable uses in cosmetics and pharmaceutical preparations. It is a terpene organic compound that has bioactive properties [5].

*C. sativum* not only has an antimicrobial effect but also has anticancer activity [6]. Cancer is the abnormal, uncontrolled growth of cells. Some types of cancer may invade other areas of the body (i.e., metastatic cancer) and require treatment with nonsurgical methods, including radiation and medications or surgical removal of the mass. Other cancers may be considered benign, which are not that dangerous and generally require no need for surgical removal of the mass [7].

Isotropic mixtures of oil, surfactant, cosurfactant, and water that form oil in water nanoemulsions under mild agitation are called self-nanoemulsifying drug delivery systems (SNEDDSs) [8]. These are considered one of the best choices to improve the delivery of nanoemulsions through the skin. This strategy will generate particle size from 20 to 200 nm [9, 10]. A nanoemulsion that is prepared by the SNEDDS method has low viscosity and transparent appearance on the skin that can improve patient compliance due to its aesthetic appeal and good skin feeling. Even if the nanoemulsion has its own advantages, we can make it more applicable by doing modifications on its viscosity to make what we call a nanoemulgel [11]. A nanoemulgel is a drug reservoir that is based on the addition of a nanoemulsion to the hydrogel matrix, such as carbomer (Carbopol), which increases thickness, reduces the interfacial tension, and develops its stability [12]. The nanoemulgel enhances the penetration of the oil into the skin. When the oily particles leave the gel matrix intact with the skin and go into its layers, then the oily particles reach their targets in the body [13, 14].

The evaluation of the antimicrobial and anticancer activities of coriander oil, in addition to the development of its nanoemulgel, will be discussed in this paper.

## 2. Materials and Methods

### 2.1. Materials

Tween 80 and Span 80 were obtained from the Al-Shams Company. Carboxyvinyl polymer (Carbopol 940) was purchased from CBC Co., Ltd., Japan. Dimethyl sulfoxide (DMSO) (Riedel De Haen, Germany) and Mueller-Hinton agar (which is produced by the Becton, Dickinson and Sparks Co. in France) were used in culture media. Hexane was purchased from Loba Chemie (India). *C. sativum* seeds were obtained from the Al-Saffarini Farm, Palestine. The plant seeds were characterized in the Pharmacy Department at An-Najah National University and kept under the voucher specimen code of Pharm-PCT-2777.

### 2.2. C. sativum Seed Oil Extraction

Firstly, 100 grams of coriander seeds were ground; then, an extraction was performed by adding 200 ml of n-hexane and 400 ml of 50% ethanol to triple distilled water. A shaker was set at 200 rpm to shake the mixture for 72 hours at room temperature. Then, a suction flask and a Buchner funnel were used to filter the mixture. After adding the filtrate into the separatory funnel, we observed two layers. The upper layer represents the organic phase containing the plant oil, while the lower layer is the aqueous layer. To evaporate the organic solvent, a rotary evaporator was used at 40°C for 1 hour. Afterwards, the completely dried seeds were stored at 25°C until used. Reextraction was done on the remaining solid material with 50 ml of hexane and 125 ml of 50% ethanol in triple distilled water. Then, the same steps were carried out as mentioned above to reextract the mixture [15].

The % yield was calculated using the following equation:

(1) $$\begin{array}{c} \%\text{yield}=\frac{M}{\text{Bm}}\times 100, \end{array}$$

where *M* is the mass of the extracted oil (g) and Bm is the initial plant biomass (g).

### 2.3. Preparation of C. sativum Oil Nanoemulgel

The formulations of *C. sativum* oil nanoemulgel were prepared by incorporating the hydrogel material (Carbopol 940) to the nanoemulsion. Therefore, nanoemulsion formulations were prepared first.

#### 2.3.1. Preparation of C. sativum Oil Nanoemulsion

After extraction, the oil was converted into a nanoemulsion by adding different weights of surfactant, cosurfactants (Tween 80 and Span 80), and coriander oil using the self-nanoemulsifying technique in order to determine a ternary phase diagram. Each formulation contained different concentrations of these three components. Afterwards, mild agitation was applied to homogenise the mixture by using the vortex for 3 minutes. The optimum formulation was chosen according to their droplet size and polydispersity index (PDI). Each formulation was self-emulsified in distilled water with mild agitation before measuring their droplet size, polydispersity index (PDI), and physical appearance [16].

#### 2.3.2. Droplet Size and PDI Analysis of C. sativum Oil Nanoemulsion

To determine the particle size and PDI, a master size analyser (Brookhaven Instruments, NanoBrook Omni, New York) was used. Self-emulsification of *C. sativum* oil nanoemulsion was carried out before applying the measuring technique, which was carried out in triplicate [17].

#### 2.3.3. Selection of C. sativum Oil Nanoemulsion Formulation

The nanoemulsion with the highest amount of *C. sativum* oil and the smallest particle size and PDI was selected to be the optimal formulation.

#### 2.3.4. Hydrogel Formulation

The preparation of the hydrogel was carried out by incorporating Carbopol 940 into water and then stirring continuously until a homogeneous mixture was obtained. 2 M NaOH was added to the mixture while stirring to adjust the hydrogel pH to 6. The mixture we prepared was then lifted for 24 hours to complete its gelation.

#### 2.3.5. C. sativum Oil Nanoemulgel Formulation

The nanoemulgel was obtained by adding Carbopol 940 with different concentrations (0.4%, 0.6%, and 0.8%) to the nanoemulsion formula that we prepared beforehand. Each formulation was mixed very well until homogeneity was reached. Then, we measured the polydispersity, droplet size, and zeta potential.

#### 2.3.6. Physical Characterization of C. sativum Nanoemulgel

There are many physical properties that we examined visually while preparing the nanoemulgel, such as consistency, spreadability, homogeneity, phase separation, and visual appearance. pH values were measured using a pH meter (CG 820, Schott Gerate GmbH, Hofheim, Germany).

#### 2.3.7. Analysis of the C. sativum Nanoemulgel Zeta Potential

For predetermination of dispersion stability and surface charge of the particles, we used the zeta potential technique, which was measured by the NanoBrook Omni. The zeta potential value was measured in triplicate to calculate the average and was graphed by showing the zeta potential versus Carbopol concentration [18].

#### 2.3.8. Rheological Measurement of Nanoemulgel

The rheological behaviour of formulations of nanoemulgels prepared with different concentrations of Carbopol 940 (0.4, 0.6, and 0.8% Carbopol as a thickening agent) was measured using 7 s size spindle. A viscometer (Brookfield DVI, USA) with shear rate range from 0 to 100 rpm was used at a temperature of 25°C. All measurements were made in triplicate. The viscosity was calculated by multiplying the density of the sample with the resulting value [17].

### 2.4. Antimicrobial Test

#### 2.4.1. Antibacterial

According to the American Type Culture Collection (ATCC), six organisms were used to perform the antibacterial test: MRSA, *Klebsiella pneumoniae*, *Escherichia coli*, *Staphylococcus aureus*, *Proteus mirabilis*, and *Pseudomonas aeruginosa*.

#### 2.4.2. Antifungal

For the antifungal test, we used *Candida albicans*.

#### 2.4.3. Culture Media

Mueller-Hinton agar (produced by the Becton, Dickinson and Sparks Co. in France) was used as the culture media, which is prepared by adding 17.5 g of acid hydrolysate of casein, 1.5 g of starch, 2 g of beef extract, and 17 g of agar per liter of purified water. Then, the components were mixed with each other until homogenous, and the mixture was heated until boiling with simple agitation to dissolve them. After this, the mixture was kept at 121°C for 20 minutes in the autoclave. The agar was coloured before being poured into sterile Petri dishes. To achieve a uniform surface and depth, we used a flat surface. Finally, the agar was stored at 4-8°C.

The antibacterial and antifungal activities were determined by using the agar diffusion process. This process was done by punching the plates containing agar in four holes (A, B, C, and D), with a 6 mm diameter. In hole A, DMSO was added only, while hole B was filled with *C. sativum* oil and DMSO. Hole C was filled with *C. sativum* nanoemulgel, and finally, hole D was filled with emulgel without *C. sativum* oil as a reference. The plates were incubated 24 hours at 37°C for the antibacterial test. On the other hand, the plates were incubated 24 hours at 25°C for the antifungal test. Measurement of the inhibition zone diameter is considered as an important step in the determination of the antibacterial and antifungal activities [16].

### 2.5. Cytotoxicity

#### 2.5.1. Cell Line and Culture

Human breast cancer MCF-7 cell line, human hepatocellular carcinoma cell line Hep3B, and human cervical epithelioid carcinoma cell line HeLa were cultured. MCF-7, Hep3B, and HeLa cells were cultured in RPMI 1640 medium (Biological Industries, USA), which was filled up with 10% fetal bovine serum, 1% penicillin/streptomycin, and 1% L-glutamine. All cells were grown in a humidified atmosphere with 5% CO_2_ at 37°C.

Cells were implanted in 96-well plates in their corresponding culture media (about 1 × 103 cells in 100 *μ*l volume/well in triplicate) and incubated for 24 hours. After that, the culture media was replaced with the same fresh corresponding culture media that contained various concentrations (300, 100, 50, and 10 *μ*g/ml) of the extract and further incubated for 72 hours. Then, the antiproliferative effect of the plant extracts was assessed by CellTiter 96®️ Aqueous One Solution Cell Proliferation (MTS) Assay according to the manufacturer's instructions (Promega Corporation, Madison, WI). At the end of the treatment, 20 *μ*l of MTS solution/100 *μ*l of media was added to each well and incubated at 37°C for 2 hours. Absorbance was measured at 490 nm [19].

## 3. Results

### 3.1. Yield of C. sativum Seed Extraction

For extraction, 1500 g of *C. sativum* seeds was prepared; 6 g of *C. sativum* oil was obtained from these seeds. As a result, the yield was 0.4%.

### 3.2. Droplet Size and PDI Analysis of C. sativum Nanoemulsion Formulations

**Figure 1:**
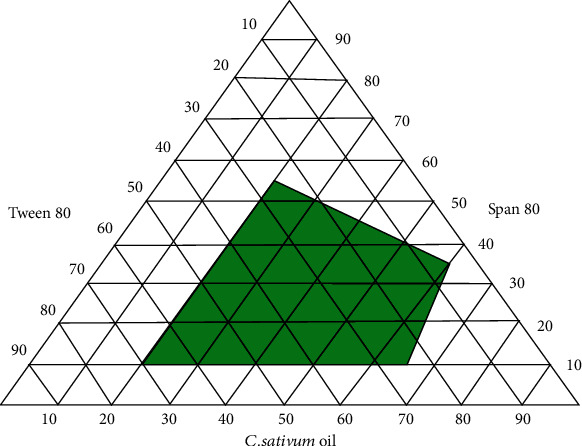
Pseudo-ternary phase diagram of *C. sativum* oil nanoemulsion.

The surfactant (Tween 80), cosurfactant (Span 80), and *C. sativum* oil with different concentrations were used to construct the ternary phase diagrams to determine the suitable formulation, which produced a nanoemulsion with a PDI < 0.3 and a droplet size < 200 nm (as shown in Figure 1).

As a comparison between the three formulations (1, 2, and 3), 45% Tween 80, 5% Span 80, and 50% *C. sativum* oil (formulation 3) were used to create the best nanoemulsion formulation, which had a PDI of 0.188 and a droplet size of 165.72 nm (as shown in Table 1).

### 3.3. C. sativum Oil Nanoemulgel Formulations

Different concentrations of Carbopol 940 (0.4, 0.6, and 0.8% *w*/*w*) were used to make nanoemulgels containing *C. sativum* oil. The gelling agent that we used was Carbopol, which provides oedematous properties to the formulation. The self-emulsification technique was used to prepare the nanoemulsion formulation by adding Tween 80 as a surfactant and Span 80 as a cosurfactant in distilled water; then, the Carbopol 940 hydrogel was added under constant stirring to form the nanoemulgel. The viscosity, droplet size, and size distribution of the nanoemulgel formulation were determined.

### 3.4. Influence of Various Carbopol Concentrations on Droplet Size and PDI of C. sativum Oil Nanoemulgel

**Figure 2:**
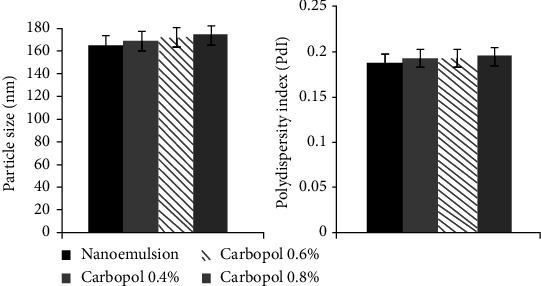
The droplet size and polydispersity index (PDI) of *C*. *sativum* oil nanoemulgel with different Carbopol concentrations.

A submicron size and narrow distribution size with low PD were shown in the mean droplet size results. The nanoemulgel was prepared with different concentrations of Carbopol 940 (0.4%, 0.6%, and 0.8%) and added to the nanoemulsion to find the optimal formulation, which has the lowest particle size and PDI. A comparison was made between the initial optimal nanoemulsion and the nanoemulgel with different concentrations of Carbopol in order to see the differences in PDI and particle size (as shown in Figure 2).

### 3.5. Sensorial Property Analysis and the Physical Characterization of C. sativum Nanoemulgel

It is all about how it is easy to take the nanoemulgel from the jar and make it spreadable enough. The more Carbopol we added, the more difficult it became to pick up the formulation from the container. So, we chose the lowest concentration of Carbopol (0.4%), rather than 0.6% and 0.8%. There was no major difference in spreadability between these different concentrations, which was good in general. The pH of the *C. sativum* nanoemulgel was 6. The optimal formulation has good spreadability, translucent colour, and an emulsification time < 30 seconds.

### 3.6. Zeta Potential Measurement of C. sativum Nanoemulgel

**Figure 3:**
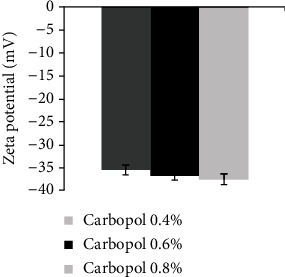
Zeta potential of *C*. *sativum* oil nanoemulgel with different Carbopol concentrations.

Based on Figure 3, the zeta potential of all the nanoemulgel formulations was below -35.

### 3.7. The Rheological Behaviour of C. sativum Oil Nanoemulgel Formulations

**Figure 4:**
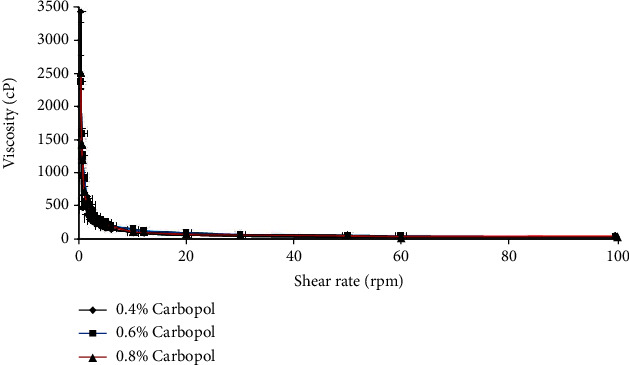
Rheological behaviour of *C*. *sativum* oil nanoemulgel with different Carbopol concentrations.

The rheological characterization evaluates the flow properties of semisolid pharmaceutical preparations to determine the effectiveness and quality of these products.  Figure 4 shows the analysis of the rheology of the nanoemulgel formulations. As the shear rate increases, there is a decrease in the viscosity, so the rheology of these formulations had a pseudoplastic behaviour.

### 3.8. Antibacterial Test

The antibacterial tests of *C. sativum* oil and nanoemulgel that we performed on different strains of gram-positive and gram-negative bacteria showed different results in comparison with control positive antibiotics and antifungals, such as ampicillin, ciprofloxacin, and fluconazole, respectively. According to the zone inhibition diameter (in cm), we noticed that the oil has effects on *K. pneumoniae*, *P. aeruginosa*, and MRSA greater than the control positive antibiotics ampicillin and ciprofloxacin, with an MIC value of 5 *μ*g/ml, 3 *μ*g/ml, and 8 *μ*g/ml, respectively, which showed the most perfect effect when the oil converted to a nanoemulgel had an MIC value of 3.75 *μ*g/ml, 2.3 *μ*g/ml, and 6.5 *μ*g/ml, respectively. On the other hand, the oil and nanoemulgel were less effective than the control positive antifungal fluconazole against *C. albicans* (as recorded in Table 2).

### 3.9. Cytotoxic Activity

In this study, we tested the anticancer effects of *C. sativum* oil and nanoemulgel in comparison with the anticancer drug doxorubicin against three types of cancer cells, namely, HeLa, Hep3B, and MCF-7 cells. HeLa cells were obtained from cervical cancer specimens, which are considered the most common type of cancer that affects women [20]. This type of cancer occurs due to many reasons, such as smoking, oral contraceptives, and human papillomavirus (HPV), which is the most common reason and considered a sexually transmitted infection [21]. However, with the improvement of screening, diagnosis, and vaccination programmes, this type of cancer is still not under control [22]. The MCF-7 cell line is derived from breast cancer, which is considered one of the biggest health problems in females and has a mortality rate that is very high worldwide [23]. Hormones, such as oestrogen, are considered one of the most common risk factors responsible for this type of cancer [24]. In addition, there are other risk factors that may play an important role, such as family history [25]. The Hep3B cells originated from hepatocellular carcinoma, which is considered a serious health problem. Most cases of hepatocellular carcinoma start with chronic hepatitis and cirrhosis caused by hepatitis B virus (HBV) and hepatitis C virus (HCV) infections, which alter the liver matrix and develop into hepatomas [26].

**Figure 5:**
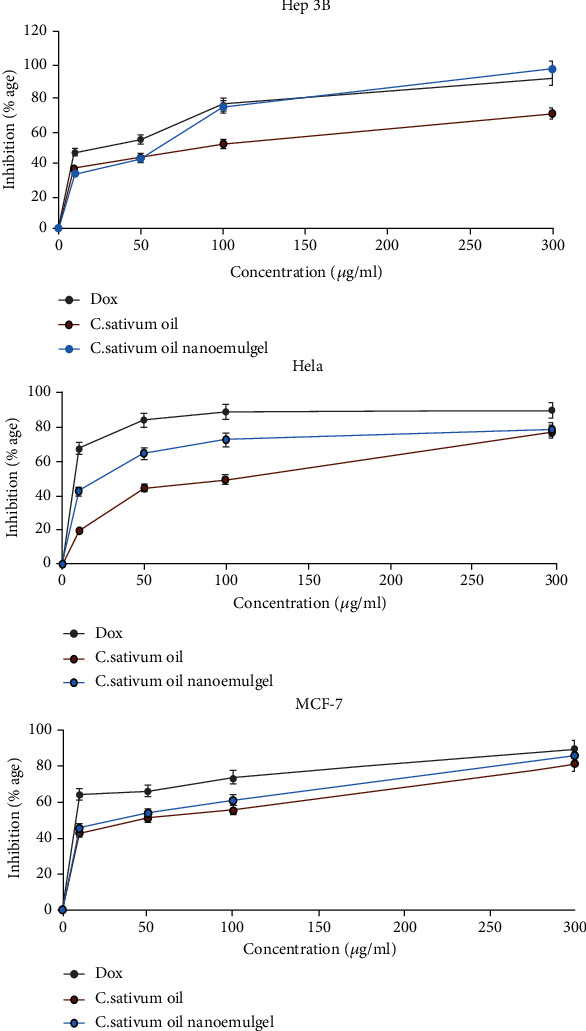
Cytotoxic effects of *C. sativum* oil and its nanoemulgel compared with doxorubicin.

After cytotoxic tests, we obtained interesting results (Figure 5), which explain the relationship between the concentration of *C. sativum* oil, its nanoemulgel, and doxorubicin plotted against the inhibition percent of cancer cell growth. When the concentrations of oil, nanoemulgel, and doxorubicin increased, the inhibition of the growth of cancer cells also increased, which means that there is an effect of oil and nanoemulgel against these cancer cells.

**Figure 6:**
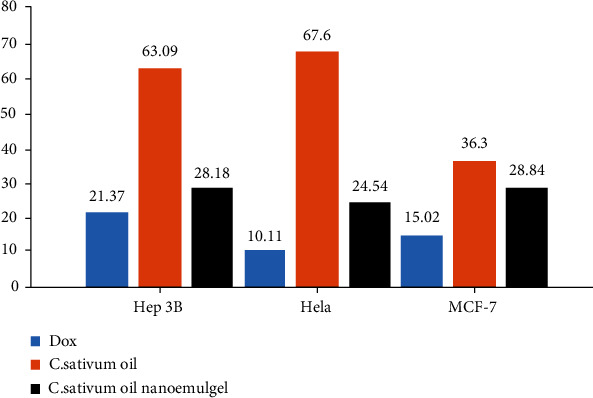
The IC_50_ values (*μ*g/ml) of *C. sativum* oil, *C. sativum* oil nanoemulgel, and doxorubicin against different cancer cell lines.

Figure 6 and Table 3 explain the IC50 of *C. sativum* oil, nanoemulgel, and doxorubicin against different types of cancer cells. When the IC_50_ decreases, the effects of them on cancer cells increase. MCF-7 cells were affected by doxorubicin, oil, and nanoemulgel with an IC_50_ of 15.02 ± 0.72 *μ*g/ml, 36.30 ± 1.17 *μ*g/ml, and 28.84 ± 0.83 *μ*g/ml, respectively, but HeLa cells were considered the most affected by doxorubicin, oil, and nanoemulgel with an IC_50_ of 10.11 ± 1.17 *μ*g/ml, 67.60 ± 1.22 *μ*g/ml, and 24.54 ± 0.95 *μ*g/ml, respectively. However, Hep3B cells were the least affected by doxorubicin, oil, and nanoemulgel with an IC_50_ of 21.37 ± 0.62 *μ*g/ml, 63.09 ± 1.32 *μ*g/ml, and 28.18 ± 0.86 *μ*g/ml, respectively. However, *C. sativum* oil and nanoemulgel were still less effective against cancer cells as compared with doxorubicin. Also, the nanoemulgel showed closer results to doxorubicin than the oil.

## 4. Discussion

As reported by Yilmaz and Borchert, adding Carbopol as a thickening agent at different concentrations did not cause a significant change in mean particle size [16, 27]. A slight increase in particle size may occur due to the increment in the viscosity [28]. At a low shear rate, increasing the concentration of Carbopol will increase the viscosity [29]. The higher the viscosity is, the more difficult the diffusion through the skin and the lower the bioavailability; therefore, we chose the lowest concentration of Carbopol, which is 0.4%, to improve the bioavailability and spreadability [30]. The rheological behaviour of the nanoemulgel was pseudoplastic, which means as the shear rate increases, there is a decrease in the viscosity [31]. The stability of the nanoemulgel depends on the magnitude of its zeta potential. The large negative and positive values of the zeta potential cause a repulsion force between particles, making the dispersion stable. Otherwise, when the zeta potential is low, the dispersion will be unstable, meaning there is no force preventing the particles from coming together. Generally, 30 mV or -30 mV is the line that separates the stability of the dispersions, while dispersions higher than 30 mV and lower than -30 mV are considered stable systems [32]. As in the results, the nanoemulgel has a value of -35 mV because of the nonionic surfactants added to the formulation that coated the system around the surface, helping to stabilize it. They did not affect the stability of the nanoemulsion in contrast to the particles [33].

The PDI plays an important role in evaluating the stability of the nanoemulgel formulation, which represents the distribution of a population's size within a given sample. If the PDI is high, the particles in the formulation become lower in homogeneity [34]. For example, the *C. sativum* nanoemulgel formulation has a PDI < 0.5, demonstrating a narrow and uniform globule size distribution [35, 36]. The formulations that have a PDI < 0.188 and droplet size around 165.72 nm are classified as high-quality formulations. In this study, a nanoemulgel with good stability is shown by a low PDI value [37].

The selection of a suitable surfactant is a very important point for the development of an appropriate nanoemulgel formulation. As a comparison between ionic and nonionic surfactants, Tween 80 (a nonionic surfactant) was selected for *C. sativum* nanoemulgel formulation, because it has a low critical micelle concentration, forms uniform and superior droplets that help with rapid absorption and release of the nanoemulgel due to a large surface area, has low toxicity compared with others, and has a low potential to cause irritation [38].

To determine the appropriate concentrations of oil, surfactant, and cosurfactant that need to be used in the formulation of the optimal self-nanoemulsifying drug delivery system (SNEDDS), you need to construct a ternary phase diagram, which is plotted to demonstrate the best formulation possible with a droplet size < 200 nm [39].

Tween 80 is a nonionic surfactant that represents 45% of our formulation. Several studies have shown that an increase in surfactant concentration usually causes a reduction in droplet particle size, which is done by using the emulsion phase inversion process [40, 41]. Furthermore, since smaller droplets have a greater surface area, they need a higher surfactant concentration to be stabilized. Literature shows that an increase in the amount of surfactant can have many effects in our formulations, such as decreasing the particle size values and increasing the interfacial area, which leads to a decrease in surface tension. This effect is due to more emulsifiers covering the surfaces of the droplets, which are formed during the process known as homogenisation [42, 43].

Recently, for the management of microbial infections, the healthcare system has gone to the use of phytomedicines. In this study, we inspected the antimicrobial activity of *C. sativum* nanoemulgel. This nanoemulgel exhibits a high zone of inhibition as compared with that of *C. sativum* oil and the positive control antibiotics ampicillin and ciprofloxacin. This bacterial inhibition is related to many reasons. Firstly, penetration of the nanoemulgel is greater than that of the oil and other medications, because the size of the particles is very small and the surface area is large, which improves the interaction of the nanoemulgel with bacteria [44]. Marslin et al. reported findings similar to ours, which showed the effect of a silver nanoparticle cream made of Withania somnifera against bacterial growth. They expected that this cream increased the bacterial inhibition zone due to an increase in its penetration. On the other hand, the study showed that the increasing contact between the bacteria and nanoemulgel was due to its packing process, which increases the concentration of nanoemulgel penetrating the bacteria [45]. Assali et al., in 2017, showed that the antibacterial activity of ciprofloxacin increased when it was converted to a single-walled carbon nanotube, which enhances its penetration and the accumulation of nanoemulgel around the bacteria, increasing its residence time [46]. Secondly, the presence of coriander oil increases the activity of nanoemulgel against gram-negative and gram-positive bacteria. According to Mandal and Mandal, a 2015 study showed that the oil has antibacterial effects against many different types of bacteria, such as *K. pneumoniae*, *S. aureus*, *P. aeruginosa*, *E. coli*, and MRSA [47]. This effect was related to its composition of linalool, *α*-pinene, *β*-pinene, p-cymene, and *γ*-terpinene [48], according to a study by Sourmaghi et al. in 2014.

In this study, we mentioned anticancer activity against three types of cells: HeLa, MCF-7, and Hep3B. This effect of nanoemulgel that we obtained related to many reasons, including the particle size of our nanoemulgel (165.72 nm), which when considered on a nanoscale suggests a particle size of 100-200 nm, so this size will facilitate its penetration into the blood supply of a tumour, giving cytotoxic effects [49]. In the study by Yue et al. in 2012, the use of PEGylated nanographene oxide as a nanocarrier increased the cytotoxic activity of graphene oxide in order to be taken up by the macrophage because of its small particle size, which causes severe inflammation and the death of tumour cells [50]. The presence of *C. sativum* oil enhanced its anticancer effect by affecting antioxidant enzymes, which led to the accumulation of H_2_O_2_ inside the cell, stopping the cell cycle, enhancing cell apoptosis, and consequently leading to the inhibition of cancer metastasis [51]. In a 2013 study, Tang et al. discussed the activity of *C. sativum* extract against the MCF-7 cell line, which contains a high number of phenolic compounds that affect the antioxidant activity, inhibiting cancer metastasis [52]. According to Freires et al., in a 2014 study on HeLa cells, *C. sativum* oil has an anticancer effect against cervical cancer by affecting proinflammatory chemokines (e.g., IL-6 and IL-8) and the protein kinase pathway [53]. In a 2020 study, Huang et al. obtained the activity of *C. sativum* extract on the Hep3B cell line, which affected its proliferation and migration, but it did not have a significant effect as in other types of cancer [54]. As mentioned previously, *C. sativum* nanoemulgel has anticancer activity but is still less than doxorubicin (an anticancer medication). This effect is due to the components of the oil and the drug delivery system that we used.

## 5. Conclusion

In this paper, *C. sativum* nanoemulgel has shown many bioactive properties, such as antimicrobial and anticancer activities, as compared to those in the crude oil and positive control medications. The improvement of the nanoemulsion, which contains coriander oil, Tween 80, and Span 80, that was prepared by the self-nanoemulsifying technique to nanoemulgel by incorporating the hydrogel material Carpobol 940 in a concentration of 0.4% leads to more penetration through the skin due to the small particle size and narrow size distribution of this formulation. The nanoemulgel has the desired rheological and physical activities. The results we obtained in this paper will be a promising step in the use of simple nanotechnology techniques in the preparation of pharmaceutical dosage forms.

## Figures and Tables

**Table 1:** The selected formulation of *C*. *sativum* oil nanoemulsion.

Formulation	Tween 80 (%)	Span 80 (%)	*C*. *sativum* oil (%)	Droplet size (nm ± SD)	PDI ± SD
1	70	10	20	189.59 ± 2.31	0.243 ± 0.09
2	60	5	35	167.71 ± 1.82	0.177 ± 0.04
3	45	5	50	165.72 ± 1.44	0.188 ± 0.07

**Table 2:** MIC values (*μ*g/ml ± 0.03) of *C. sativum* oil and *C. sativum* oil nanoemulgel compared with ampicillin, ciprofloxacin, and fluconazole antibiotics.

Microorganisms	*C. sativum* oil	*C. sativum* oil nanoemulgel	Ampicillin	Ciprofloxacin	Fluconazole
*S. aureus* (ATCC 25923)	9	8	6.25	0.78	—
MRSA	8	6.5	32	12.5	—
*E. coli* (ATCC 25922)	5.5	5	3.12	0.78	—
*P. vulgaris* (ATCC 8427)	8	7	3.25	0.06	—
*K. pneumoniae* (ATCC 13883)	5	3.75	12.5	0.06	—
*P. aeruginosa* (ATCC 9027)	3	2.3	100	3.12	—
*C. albicans* (ATCC 90028)	6	4.5	—	—	3.12

**Table 3:** The IC_50_ values (*μ*g/ml) of *C. sativum* oil, *C. sativum* oil nanoemulgel, and doxorubicin against different cancer cell lines.

	Hep3B	Hela	MCF-7
*C. sativum* oil IC_50_ (*μ*g/ml)	63.09 ± 1.32	67.60 ± 1.22	36.30 ± 1.17
*C. sativum* oil nanoemulgel IC_50_ (*μ*g/ml)	28.18 ± 0.86	24.54 ± 0.95	28.84 ± 0.83
Doxorubicin IC_50_ (*μ*g/ml)	21.37 ± 0.62	10.11 ± 1.17	15.02 ± 0.72

## Data Availability

All the necessary data are included in the manuscript.

## References

[1] Patra J. K., Das G., Fraceto L. F. (2018). Nano based drug delivery systems: recent developments and future prospects. *Journal of Nanobiotechnology*.

[2] Buya A. B., Beloqui A., Memvanga P. B., Préat V. (2020). Self-nano-emulsifying drug-delivery systems: from the development to the current applications and challenges in oral drug delivery. *Pharmaceutics*.

[3] Momin A. H., Acharyaand S. S., Gajjar A. V. (2012). Coriandrum sativum-review of advances in phytopharmacology. *International Journal of Pharmaceutical Sciences and Research*.

[4] Aelenei P., Rimbu C. M., Guguianu E. (2019). Coriander essential oil and linalool - interactions with antibiotics against gram-positive and gram-negative bacteria. *Letters in Applied Microbiology*.

[5] Pereira I., Severino P., Santos A. C., Silva A. M., Souto E. B. (2018). Linalool bioactive properties and potential applicability in drug delivery systems. *Colloids and Surfaces B: Biointerfaces*.

[6] Sathishkumar P., Preethi J., Vijayan R. (2016). Anti-acne, anti-dandruff and anti-breast cancer efficacy of green synthesised silver nanoparticles using Coriandrum sativum leaf extract. *Journal of Photochemistry and Photobiology B: Biology*.

[7] Wang J. J., Lei K. F., Han F. (2018). Tumor microenvironment: recent advances in various cancer treatments. *European Review for Medical and Pharmacological Sciences*.

[8] Date A., Nagarsenker M. (2007). Design and evaluation of self-nanoemulsifying drug delivery systems (SNEDDS) for cefpodoxime proxetil. *International Journal of Pharmaceutics*.

[9] Algahtani M. S., Ahmad M. Z., Ahmad J. (2020). Nanoemulgel for improved topical delivery of retinyl palmitate: formulation design and stability evaluation. *Nanomaterials*.

[10] Buya A. B., Ucakar B., Beloqui A., Memvanga P. B., Préat V. (2020). Design and evaluation of self-nanoemulsifying drug delivery systems (SNEDDSs) for senicapoc. *International Journal of Pharmaceutics*.

[11] Date A. A., Desai N., Dixit R., Nagarsenker M. (2010). Self-nanoemulsifying drug delivery systems: formulation insights, applications and advances. *Nanomedicine*.

[12] Dhawan B., Aggarwal G., Harikumar S. (2014). Enhanced transdermal permeability of piroxicam through novel nanoemulgel formulation. *International Journal of Pharmaceutical Investigation*.

[13] Aman R. M., Abu Hashim I. I., Meshali M. M. (2020). Novel clove essential oil nanoemulgel tailored by Taguchi’s model and scaffold-based nanofibers: phytopharmaceuticals with promising potential as cyclooxygenase-2 inhibitors in external inflammation. *International Journal of Nanomedicine*.

[14] Mao Y., Chen X., Xu B. (2019). Eprinomectin nanoemulgel for transdermal delivery against endoparasites and ectoparasites: preparation,in vitroandin vivoevaluation. *Drug Delivery*.

[15] Eid A. M., Jaradat N. A., Elmarzugi N. A. (2019). Anti-microbial and free radical scavenging activities of nigella sativa colloidal-emulgel. *Letters in Drug Design & Discovery*.

[16] Eid A. M., Jaradat N. A., al-Masri M. (2020). Development and antimicrobial evaluation of Eruca sativa oil nanoemulgel with determination of the oil antioxidant, sun protection factor and elastase inhibition. *Current Pharmaceutical Biotechnology*.

[17] Eid A. M., Istateyeh I., Salhi N., Istateyeh T. (2019). Antibacterial activity of fusidic acid and sodium fusidate nanoparticles incorporated in pine oil nanoemulgel. *International Journal of Nanomedicine*.

[18] Đorđević S. M., Cekić N. D., Savić M. M. (2015). Parenteral nanoemulsions as promising carriers for brain delivery of risperidone: Design, characterization and _in vivo_ pharmacokinetic evaluation. *International Journal of Pharmaceutics*.

[19] Eid A. M., Hawash M. (2021). Biological evaluation of safrole oil and safrole oil nanoemulgel as antioxidant, antidiabetic, antibacterial, antifungal and anticancer. *BMC Complementary Medicine and Therapies*.

[20] Vu M., Yu J., Awolude O. A., Chuang L. (2018). Cervical cancer worldwide. *Current Problems in Cancer*.

[21] Tsikouras P., Zervoudis S., Manav B. (2016). Cervical cancer: screening, diagnosis and staging. *Journal of B.U.ON*.

[22] Canavanand T. P., Doshi N. R. (2000). Cervical cancer. *American Family Physician*.

[23] Li J., Guo Y., Duan L. (2017). AKR1B10 promotes breast cancer cell migration and invasion via activation of ERK signaling. *Oncotarget*.

[24] Tian J. M., Ran B., Zhang C. L., Yan D. M., Li X. H. (2018). Estrogen and progesterone promote breast cancer cell proliferation by inducing cyclin G1 expression. *Brazilian Journal of Medical and Biological Research*.

[25] Harris J. R., Lippman M. E., Veronesi U., Willett W. (1992). Breast cancer. *New England Journal of Medicine*.

[26] Thorgeirsson S. S., Grisham J. W. (2002). Molecular pathogenesis of human hepatocellular carcinoma. *Nature Genetics*.

[27] Yilmaz E., Borchert H. H. (2006). Effect of lipid-containing, positively charged nanoemulsions on skin hydration, elasticity and erythema--an in vivo study. *International Journal of Pharmaceutics*.

[28] Chakraborty S., Khandai M., Sharma A. (2010). Preparation, in vitro and in vivo evaluation of algino-pectinate bioadhesive microspheres: an investigation of the effects of polymers using multiple comparison analysis. *Acta Pharmaceutica*.

[29] Eid A. M., El-Enshasy H. A., Aziz R., Elmarzugi N. A. (2014). Preparation, characterization and anti-inflammatory activity of Swietenia macrophylla nanoemulgel. *Journal of Nanomedicine and Nanotechnology*.

[30] Boddupalli B. M., Mohammed Z. N., Nath R. A., Banji D. (2010). Mucoadhesive drug delivery system: an overview. *Journal of Advanced Pharmaceutical Technology & Research*.

[31] Sharma S., Sharma A. D., Naseer M., Singh R. (2011). Formulation and evaluation of self emulsifying drug delivery system of ibuprofen using castor oil. *International Journal of Pharmaceutical Sciences Research*.

[32] Arriaga L. R., Drenckhan W., Salonen A. (2012). On the long-term stability of foams stabilised by mixtures of nano-particles and oppositely charged short chain surfactants. *Soft Matter*.

[33] Salim N., Basri M., Abdullah D. K., Basri H. (2011). Phase behaviour, formation and characterization of palm-based esters nanoemulsion formulation containing ibuprofen. *Journal of Nanomedicine and Nanotechnology*.

[34] Avachat A. M., Patel V. G. (2015). Self nanoemulsifying drug delivery system of stabilized ellagic acid- phospholipid complex with improved dissolution and permeability. *Saudi Pharmaceutical Journal*.

[35] Balakumar K., Raghavan C. V., Abdu S. (2013). Self nanoemulsifying drug delivery system (SNEDDS) of rosuvastatin calcium: design, formulation, bioavailability and pharmacokinetic evaluation. *Colloids and Surfaces. B, Biointerfaces*.

[36] Shakeel F., Haq N., Alanazi F. K., Alsarra I. A. (2014). Polymeric solid self-nanoemulsifying drug delivery system of glibenclamide using coffee husk as a low cost biosorbent. *Powder Technology*.

[37] Nepal P. R., Han H. K., Choi H. K. (2010). Preparation and _in vitro_ - _in vivo_ evaluation of Witepsol ^®^ H35 based self-nanoemulsifying drug delivery systems (SNEDDS) of coenzyme Q_10_. *European Journal of Pharmaceutical Sciences*.

[38] Azeem A., Rizwan M., Ahmad F. J. (2009). Nanoemulsion components screening and selection: a technical note. *AAPS PharmSciTech*.

[39] Mayer S., Weiss J., McClements D. J. (2013). Vitamin E-enriched nanoemulsions formed by emulsion phase inversion: factors influencing droplet size and stability. *Journal of Colloid and Interface Science*.

[40] Bilbao-Sáinz C., Avena-Bustillos R. J., Wood D. F., Williams T. G., McHugh T. H. (2010). Nanoemulsions prepared by a low-energy emulsification method applied to edible films. *Journal of Agricultural and Food Chemistry*.

[41] Ostertag F., Weiss J., McClements D. J. (2012). Low-energy formation of edible nanoemulsions: factors influencing droplet size produced by emulsion phase inversion. *Journal of Colloid and Interface Science*.

[42] Arbain N. H., Salim N., Wui W. T., Basri M., Rahman M. B. A. (2018). Optimization of quercetin loaded palm oil ester based nanoemulsion formulation for pulmonary delivery. *Journal of Oleo Science*.

[43] Li Y., Zhang Z., Yuan Q., Liang H., Vriesekoop F. (2013). Process optimization and stability of D-limonene nanoemulsions prepared by catastrophic phase inversion method. *Journal of Food Engineering*.

[44] Nirmala M. J., Mukherjee A., Chandrasekaran N. (2014). Retraction note: design and formulation technique of a novel drug delivery system for azithromycin and its anti-bacterial activity against Staphylococcus aureus. *AAPS PharmSciTech*.

[45] Marslin G., Selvakesavan R. K., Franklin G., Sarmento B., Dias A. C. (2015). Antimicrobial activity of cream incorporated with silver nanoparticles biosynthesized from Withania somnifera. *International Journal of Nanomedicine*.

[46] Assali M., Zaid A. N., Abdallah F., Almasri M., Khayyat R. (2017). Single-walled carbon nanotubes-ciprofloxacin nanoantibiotic: strategy to improve ciprofloxacin antibacterial activity. *International Journal of Nanomedicine*.

[47] Mandal S., Mandal M. (2015). Coriander (Coriandrum sativum L.) essential oil: Chemistry and biological activity. *Asian Pacific Journal of Tropical Biomedicine*.

[48] Sourmaghi M. H., Kiaee G., Golfakhrabadi F., Jamalifar H., Khanavi M. (2015). Comparison of essential oil composition and antimicrobial activity of Coriandrum sativum L. extracted by hydrodistillation and microwave-assisted hydrodistillation. *Journal of Food Science and Technology*.

[49] Pei X., Zhu Z., Gan Z. (2020). PEGylated nano-graphene oxide as a nanocarrier for delivering mixed anticancer drugs to improve anticancer activity. *Scientific Reports*.

[50] Yue H., Wei W., Yue Z. (2012). The role of the lateral dimension of graphene oxide in the regulation of cellular responses. *Biomaterials*.

[51] Al-Snafi A. E. (2016). A review on chemical constituents and pharmacological activities of Coriandrum sativum. *IOSR Journal of Pharmacy*.

[52] Tang E. L., Rajarajeswaran J., Fung S. Y., Kanthimathi M. S. (2013). Antioxidant activity of Coriandrum sativum and protection against DNA damage and cancer cell migration. *BMC Complementary and Alternative Medicine*.

[53] Freires I. . A., Murata R. M., Furletti V. F. (2014). Coriandrum sativum L. (Coriander) essential oil: antifungal activity and mode of action on Candida spp., and molecular targets affected in human whole-genome expression. *PLoS One*.

[54] Huang H., Nakamura T., Yasuzawa T., Ueshima S. (2020). Effects of Coriandrum sativum on migration and invasion abilities of cancer cells. *Journal of Nutritional Science and Vitaminology*.

